# The validity of the diagnosis of inflammatory arthritis in a large population-based primary care database

**DOI:** 10.1186/1471-2296-14-79

**Published:** 2013-06-07

**Authors:** Markus MJ Nielen, Jennie Ursum, François G Schellevis, Joke C Korevaar

**Affiliations:** 1 NIVEL (Netherlands Institute for Health Services Research), P.O. Box 1568, Utrecht, 3500BN, The Netherlands; 2 Department of General Practice & Elderly Care Medicine EMGO Institute for Health and Care Research, VU University Medical Center, Amsterdam, The Netherlands

**Keywords:** Validity, Inflammatory arthritis, General practice, EMRs

## Abstract

**Background:**

Large population-based databases based on electronic medical records (EMRs) of patients in primary care are a useful data source to investigate morbidity and health care utilization. Diagnoses recorded in EMRs are doctor-defined, but their validity can be disputed. In this study we investigated the validity of the diagnosis inflammatory arthritis (IA), a group of chronic rheumatic diseases, including rheumatoid arthritis, psoriatic arthritis and ankylosing spondylitis, in primary care based EMRs.

**Methods:**

In five general practices, participating in the Netherlands Information Network of General Practice (LINH), EMRs of 219 patients with a diagnostic code of IA were systematically reviewed on characteristics which are not routinely extracted for the LINH database. The diagnosis IA was confirmed when we found, based on a correspondence with a medical specialist, the following diagnoses in the free text fields of the EMR: oligoarthritis, polyarthritis, rheumatoid arthritis and/or spondyloarthropathy. These results were used to determine the validity of the diagnosis IA in EMRs and to develop an algorithm to improve diagnostic validity.

**Results:**

From the 219 patients diagnosed as IA in the database, the diagnosis IA was confirmed in 155 patients (70.8%). The algorithm, which resulted in a group of patients with as many as possible confirmed IA-diagnosed patients without excluding too many patients from our dataset, was when patients fulfilled at least one of the following three criteria: 1) a repeat prescription for a disease-modifying antirheumatic drug (DMARD) and/or biological agent, 2) ≥ four contacts or one episode with a diagnostic code for IA, combined with at least two IA-related prescriptions (excluding DMARDs/biological agents), and 3) age at diagnosis ≥ 61 years. After applying this algorithm, the percentage of correctly diagnosed IA patients increased from 71% to 78% reducing the size of our study population by 36%.

**Conclusions:**

Based on additional diagnostic information, the diagnosis IA from EMRs of patients in primary care is sufficiently valid when using the proposed algorithm. After applying the algorithm, the percentage of correctly diagnosed IA patients increased from 71% to 78%.

## Background

A major advantage of using electronic medical records (EMRs) from general practitioners (GPs) is the possibility to study large numbers of patients over a long period. This makes it, for example, possible to investigate diseases with a low prevalence, or monitor disease course over time. Furthermore, patients from primary care, in countries where the GP acts as gatekeeper and where inhabitants are listed in a practice, are representative for the total population, including the whole range of disease severity. Cohorts in secondary care often contain only patients with more severe disease-stage, which makes it, compared with primary care, more difficult to generalize results to the total patient population. Finally, in case of fixed patient lists, control patients can be easily selected for case–control studies from the same practice as the cases, which diminishes the possible bias due to practice variation.

In the Netherlands, all inhabitants are listed with a general practice and generally the GP is the first professional to be consulted for health problems. As in many countries, GPs have a gatekeeper role for access to specialized care, and therefore their EMRs include a complete record of all morbidity of their patients using a uniform methodology. According to the guidelines of the Dutch College of GPs, GPs are expected to record diagnostic information from their patients routinely in EMRs, using the International Classification of Primary Care version 1 (ICPC-1) [1].

Despite these advantages, the use of EMRs from GPs for medical research has a number of disadvantages. First, EMRs only contain routinely recorded information about patients who visited their GP for complaints and data are usually not collected in a systematic way. For many patients, who do not visit their GP on a regular basis, risk factors such as smoking status, body mass index or family history of cardiovascular diseases are unknown. Secondly, the diagnoses of diseases, although doctor-defined, are not always valid. It is likely that diagnoses of chronic diseases that are mainly treated by a GP or diseases that are easily identified, for example with a diagnostic test with a high sensitivity, like diabetes mellitus or chronic obstructive pulmonary disease, are sufficiently valid within an EMR of a GP. However, for diseases with a less clear diagnostic test or diseases which could be easily mixed up with other diseases, the diagnosis is not always correct.

Inflammatory arthritis (IA), defined as a group of chronic rheumatic diseases, including rheumatoid arthritis, psoriatic arthritis and ankylosing spondylitis, is an example of a difficult diagnosis to make. Moreover, it can be difficult for a GP to distinguish IA from other rheumatic diseases like osteoarthritis (OA) and gout at disease onset. Therefore, it is expected that a registered IA diagnosis in an EMR of the GP is not always valid.

When carrying out studies in IA patients, such a study will only provide reliable information if the validity of the diagnosis can be confirmed. The aim of this study is therefore 1) to investigate the validity of the diagnosis IA in primary care based records and 2) to develop an algorithm to improve the validity of this diagnosis.

## Methods

### Study population

The database of the Netherlands Information Network of General Practice (LINH) includes routinely extracted data from EMRs from on average 85 general practices with about 350,000 listed patients since 2001 (http://www.linh.nl). Five general practices participating in LINH were visited in 2011 to collect extra diagnostic information. We selected practices which were representative for all LINH practices in terms of degree of urbanisation, since patient populations can differ between a small town and a large city. In these general practices, all patients of 30 years and older who were ever registered between 2001 and 2010 with a diagnostic code of IA (ICPC-1 code L88) in the LINH database in 2010 were systematically reviewed on characteristics which are not routinely extracted for the LINH database: free text regarding contacts, prescriptions, medical history, referrals and correspondence with medical specialists. The studied population included incident as well as prevalent cases with the whole range of disease duration. Only patients of 30 years and older were included in the study to exclude patients with juvenile arthritis.

The study was carried out according to Dutch legislation on privacy. The privacy regulation of the study is approved by the Dutch Data Protection Authority. According to Dutch legislation, neither obtaining informed consent nor approval by a medical ethics committee was obligatory for observational studies. In concordance with the GPs, EMRs were reviewed in the participating practices and data were analysed anonymously.

### Validity of the diagnosis inflammatory arthritis

The diagnosis IA was confirmed when we found, based on a correspondence with a medical specialist, the following diagnoses in the free text fields of the EMR: oligoarthritis, polyarthritis, rheumatoid arthritis and/or spondyloarthropathy. Charts were reviewed manually and systematically for letters from medical specialists and other correspondence (for instance by telephone) in the free text fields. The validity of the diagnosis IA in EMRs was determined by calculating the percentage of patients in whom the diagnosis IA was confirmed after chart review.

### Algorithm to improve validity of the diagnosis inflammatory arthritis

Since we expected that a registered IA diagnosis in an EMR of the GP is not always valid, an algorithm was developed to improve the validity of the diagnosis IA in the LINH database. All routinely available information in our database was used to develop this algorithm to distinguish IA from non-IA in patients with an ICPC-1 code L88, including the following parameters: age, gender, number of L88-related visits or an L88-related episode with the GP, prescriptions with the IA diagnostic code (excluding DMARDs and biological agents) and repeat prescriptions of disease-modifying antirheumatic drugs (DMARDs) and/or biological agents. A disease episode is the period between the first and last contact of the patient with the GP for a specific health problem, which can include multiple contacts. A repeat prescription is defined as a prescription issued without a consultation with the GP. In general, a medical specialist prescribes a DMARD, which is not recorded in the EMR of the GP, which can be followed by a repeat prescription by the GP. Based on these available parameters, we defined three criteria. The first criterion was the use of IA medication, defined as ‘a repeat prescription for a disease-modifying antirheumatic drug (DMARD) or biological agent’. The second criterion was based on IA-related consultations and prescriptions, defined as ‘at least four contacts or one episode with a diagnostic code for IA (L88), combined with at least two prescriptions with the IA diagnostic code (excluding DMARDs/biological agents)’. Finally, the third criterion was based on age at the time of diagnosis (i.e. age at the moment of the first L88-coded consultation). The cut-off for age was set at 61 years old, since this was the mean age at time of diagnosis. Patients above 61 years old were more likely to have other diagnoses than IA, such as osteoarthritis. These three criteria were used to develop an algorithm to define a group of patients with as many as possible confirmed IA-diagnosed patients without excluding the majority of the patients from our dataset including many with an IA diagnosis.

## Results

In the five participating general practices, 219 patients were identified with a recorded IA diagnosis (ICPC-1 code L88) in the LINH database. At the time of the first contact with ICPC-1 code L88, their mean age was 58 years (SD=15) and 64% was female. The diagnosis IA was confirmed after chart review in 155 patients (70.8%).

Three criteria were used to develop an algorithm to define a group of patients with as many as possible confirmed IA-diagnosed patients without excluding too many patients from our dataset: 1) a repeat prescription for a DMARD and/or biological agent, 2) ≥ four contacts or one episode with a diagnostic code for IA, combined with at least two IA-related prescriptions (excluding DMARDs/biological agents), and 3) age at diagnosis ≤ 61 years. The combination of consultations and prescriptions resulted in the highest percentage confirmed IA diagnoses. The number of L88-coded patients in the LINH database who fulfilled each criterion and all combinations of these criteria are shown in Table 1.

**Table 1:** Number of L88-coded patients in the LINH database who fulfilled combinations of criteria of the algorithm to distinguish IA from non-IA (n=219)

	**Number of patients (n=219) who fulfilled criteria (%)**	**Confirmed diagnosis of IA?**	
		**Yes**	**No**
***Without using an algorithm***	---	155 (70.8%)	64
***Algorithm based on one criterion:***			
Criterion 1: Repeat prescription DMARDs/biological agents	47 (21.5%)	43 (91.5%)	4
Criterion 2: Contact with GP and IA-related prescription^1^	49 (22.4%)	44 (89.8%)	5
Criterion 3: Age 30–61 years at first diagnosis	105 (47.9%)	80 (76.2%)	25
***Algorithm based on two criteria:***			
Criterion 1 or 2	73 (33.3%)	64 (87.7%)	9
Criterion 1 or 3	127 (58.0%)	101 (79.5%)	26
Criterion 2 or 3	132 (60.3%)	102 (77.3%)	30
***Algorithm based on three criteria:***			
All 3 criteria	9 (4.1%)	9 (100%)	0
2 out of 3 criteria	52 (23.7%)	49 (94.2%)	3
1 out of 3 criteria	140 (63.9%)	109 (77.9%)	31

^1^ ≥ 4 L88 ICPC-1 codes at a contact and ≥ 2 L88 ICPC-1 codes at a prescription OR ≥ 1 L88 ICPC-1 code at an episode and ≥ 2 L88 ICPC-1 codes at a prescription.

Applying criterion 1 resulted in a population with the highest number of patients with a confirmed IA diagnosis (91.5%), but the lowest number of included patients (21.5%). On the other hand, using criterion 3 increases the number of included patients (47.9%), but reduces the number of patients with a confirmed IA diagnosis (76.2%). Combining two of the three defined criteria, resulted in general in lower validity of the IA diagnosis (i.e. lower number of confirmed IA diagnoses), but a higher number of patients who fulfil the criteria, compared with using only one criterion. Validity varied between 77.3% and 87.7% with 33.3% to 60.3% included patients. Finally, using all three criteria in an algorithm resulted in 100% validity when patients needed to fulfil all three criteria. However, only 9 out of 219 patients (4.1%) fulfilled all three criteria. The algorithm, which resulted in a group of patients with as many as possible confirmed IA-diagnosed patients without excluding too many patients from our dataset, was when patients fulfilled at least one of the three criteria. Applying this algorithm resulted in a group of 140 patients (excluding 36.1% of all patients) with a validity of the diagnosis IA of 78%.

## Discussion

After chart review, the diagnosis IA from EMRs of 219 patients was confirmed in 155 patients (71%). All routinely available information in our database was used to develop an algorithm to identify IA patients. After applying the algorithm, the percentage of correctly diagnosed IA patients increased from 71% to 78%.

To our knowledge this is the first validation study of the diagnosis inflammatory arthritis in EMRs from GPs. The validity of the diagnosis rheumatoid arthritis, based on other classification systems, such as the International Classification of Diseases (ICD), has already been determined in several cohorts [2-5]. These studies showed sufficient validity of the diagnosis, especially after using algorithms to define patients who are most likely to have the diagnosis of a rheumatic disease; the range between 72% and 91% is in line with our results. In a systematic review, Herrett *et al.* studied the validity of 183 different diagnoses in primary care and found a median of 80% confirmed diagnoses in the group musculoskeletal disorders [6]. This is in line with the results of our study after applying our algorithm.

After applying our algorithm, in about 20% of the patient group with a recorded IA diagnosis this could not be confirmed. In these patients, besides an ICPC-1 L88-related contact, no additional information was found in the free text fields regarding contacts, prescriptions, medical history, referrals and correspondence with medical specialists to confirm the diagnosis IA. The diagnosis IA could have been registered once due to suspicion for a rheumatic disease and changed later in a diagnosis gout or osteoarthritis. Despite this percentage of false positives, we think that EMRs from GPs are still a useful data source with sufficient power for studying morbidity and health care utilization in IA or OA patients. Moreover, it is often clear in which direction results are biased when including a small proportion with less severe diseases. Including patients without an IA diagnosis into the study population will result in an overestimation of both the incidence and prevalence rates of IA. On the other hand, comorbidity and health care utilization will be underestimated. Patients without a diagnosis IA are less likely to develop comorbidity or to use certain medication, resulting in lower rates compared with non-biased study populations. This limitation should always be discussed together with the advantages of using EMRs for medical research, such as the use of large patient groups and having a representative study population with the whole range of disease severity.

This study has some limitations. First, we used a sample of patients from just five general practices. We did not validate our algorithm in another set of general practices. Second, the diagnosis of the medical specialist was used as gold standard. It is unclear to what extent medical specialists made wrong diagnoses. Moreover, we only sought to confirm the IA diagnosis recorded by the GP; the number of true IA patients without a diagnosis IA (false negatives) in the EMRs of the GP was not established. Finally, given the low percentage of patients who did fulfil the first criterion of the algorithm, it is likely that a number of DMARD users is incorrectly excluded. A DMARD is, in most cases, prescribed by a rheumatologist, followed by a repeat prescription of the GP. These repeat prescriptions are not always registered in the EMR of the GP. Since DMARD users are the more severe IA patients, it is expected that these patients fulfil the second criterion of our algorithm, including GP consultations and prescriptions.

## Conclusions

Based on additional diagnostic information, the diagnosis IA from EMRs of patients in primary care is sufficiently valid when using the proposed algorithm. After applying the algorithm, the percentage of correctly diagnosed IA patients increased from 71% to 78%.

## Competing interests

The authors declare that they have no competing interests.

## Authors’ contributions

MMJN and JU performed the study according to protocol and analysed the data. MMJN wrote the manuscript. FGS and JCK supervised the study and developed the study protocol. All authors read and approved the final manuscript.

## Pre-publication history

The pre-publication history for this paper can be accessed here:

http://www.biomedcentral.com/1471-2296/14/79/prepub
